# Genomic features and pathogenic potential of *Streptococcus agalactiae* isolated from bovine clinical mastitis

**DOI:** 10.5455/javar.2025.l874

**Published:** 2025-03-24

**Authors:** Jayedul Hassan, Abdus Sattar Bag, Susmita Karmakar, Kishor Sosmith Utsho, Wohab Ali, Ajran Kabir, Tanvir Rahman

**Affiliations:** Department of Microbiology and Hygiene, Bangladesh Agricultural University, Mymensingh, Bangladesh

**Keywords:** *S. agalactiae*, clinical mastitis, genomic features, Bangladesh

## Abstract

**Objective:**

The goal of this study is to describe the genome of *Streptococcus agalactiae* that was found in clinical mastitis in cattle in Bangladesh. This work will show how strong the bacteria are and how important they are for public health.

**Materials and Methods:**

Whole genome sequencing (WGS) was performed using the Illumina MiSeq platform, followed by comprehensive analysis with various bioinformatic tools to identify key genomic features.

**Results:**

WGS revealed that the isolates are closely related, belonging to sequence type ST4, a rare type previously identified in both human and animal hosts. The isolates possess 44 virulence-related genes linked to adherence, capsule biogenesis, enzyme production, immunoreactive antigens, protease, and cytolysin production. They also carry two pilus islands (PIs), PI-1 and PI-2b, which are often associated with invasive diseases. PI-2b proteins are key targets for vaccine development against Group B Streptococcus (GBS). The isolates belong to serotype Ia and carry the gbs2018-2 variant, indicating their adaptability to a wide range of hosts, including humans and animals. These virulence factors are critical for understanding *S. agalactiae’*s pathogenicity and developing vaccines against its infections. Additionally, the isolates harbor antimicrobial resistance genes conferring resistance to glycopeptides (*vanT*, *vanY*), macrolides (*mreA*), peptides (*mprF*), penicillins and *β*-lactams (*pbp*), and aminoglycosides. Source tracking via the BacWGSTdb website identified these isolates as closely related to human pathogens, indicating their zoonotic potential.

**Conclusion:**

These results suggest that *S. agalactiae* could be a zoonotic pathogen. This highlights the need for ongoing genomic surveillance to fully understand how it causes disease and come up with effective ways to control it.

## Introduction

*Streptococcus agalactiae, *widely known as Group B Streptococcus (GBS), is a gram-positive pathogenic bacterium that causes subclinical and clinical mastitis in dairy cattle, leading to substantial economic losses in the dairy sector [1,2]*.* Mastitis, an inflammation of the mammary gland, adversely affects milk production and quality, thereby impacting the dairy industry on a global scale. The pathogenic nature of *S. agalactiae* in bovine mastitis stems from its capacity to adhere to and invade mammary epithelial cells, evade the host’s immune system, and produce various virulence factors (VFs) that intensify the infection [3].

Beyond its veterinary significance, *S. agalactiae* is a major human pathogen. It is responsible for severe infections such as pneumonia, neonatal sepsis, endocarditis, meningitis, and other serious diseases in humans, particularly affecting newborns, the elderly, and pregnant women [4–6]. The dual-host nature of *S. agalactiae* raises concerns about its zoonotic potential and the possibility of cross-species transmission. Indirect data suggests that *S. agalactiae* is transmitted from cattle to humans, posing a significant public health concern [7].

In our previous study, we reported the occurrence of *S. agalactiae* in clinical mastitis in Bangladesh, highlighting the need for further investigation into its genomic characteristics [8]. In this study, we aimed to provide a comprehensive analysis of the *S. agalactiae* genomes, focusing on the elucidation of virulence determinants and the public health significance of this pathogen by WGS and analysis.

Considering the findings of previous studies, our research concentrates on several key parameters considered in the molecular epidemiology and genomic diversity of *S. agalactiae*, such as virulence and antimicrobial resistance (AMR) determinants, sequence types (STs), molecular serotypes, and mobile genetic elements including plasmids, phages, and insertion sequences. Based on our research, this is the inaugural study detailing the genome sequencing and comprehensive analysis of *S. agalactiae* from cases of clinical mastitis in cattle in Bangladesh. This research not only fills a critical knowledge gap but also provides valuable insights into the pathogenic potential and public health implications of *S. agalactiae*.

## Materials and Methods

### Ethical approval

This study does not involve any animals or living beings. Thus, no ethical approval was required.

### Bacterial strains

*Streptococcus agalactiae* was revived from our repository previously isolated from cattle with clinical mastitis [8]. The isolates were recovered from the same dairy farm and were multidrug-resistant with variable resistance patterns.

### Sequencing and assembly

Whole genome sequencing (WGS) was conducted on an Illumina NextSeq 2000 platform (Illumina, CA, USA) at the Child Health Research Foundation), Dhaka. Sequence assembly was performed on the Galaxy platform [9]. FASTQ reads were trimmed to filter out low-quality reads on Trimmomatic (Galaxy Version 0.38), followed by assembly by Unicycler (Galaxy Version 0.4.8.0). Following assembly, the genome underwent annotation and ioinformatics analysis to identify STs, virulence genes, and AMR genes (ARGs).

### Annotation and bioinformatics analysis

To identify the functional features, annotation was performed by Prokka (Galaxy Version 1.14.6), Rapid Annotation using Subsystem Technology (https://rast.nmpdr.org/rast.cgi), and the NCBI Prokaryotic Genome Annotation Pipeline. Furthermore, the genomes were analyzed with the Roary Pan Genome pipeline for orthologous genes in *S. agalactiae* and constructed a gene presence-absence matrix [10]. Additionally, the STs of the *S. agalactiae* genome sequences were determined using the PubMLST website (pubmlst.org). To explore the close relatives, the *S. agalactiae* sequences were investigated on the BacWGSTdb website (http://bacdb.cn/BacWGSTdb/analysis_single.php) based on core genome multilocus sequence typing (cgMLST). For circular visualization of the *S. agalactiae* genomes and identification of CRISPR-Cas Proksee tools (https://Proksee.ca) were used.

### Mobile genetic elements (plasmids, phages, IS elements)

PlasmidFinder (https://cge.food.dtu.dk/services/PlasmidFinder/) and the PHASTER web server (https://phaster.ca) were used for plasmids and prophages, respectively. For identification of IS elements, the annotated genome was searched on the Isfinder database using default parameters (https://www-is.biotoul.fr/blast.php) as well as CLC Genomic Workbench 22 for manual curation (Qiagen, Germany).

### AMR and VF genes

Genes conferring AMR were identified through the Comprehensive Antibiotic Resistance Database (https://card.mcmaster.ca/). For profiling virulence genes, the virulence factor database (www.mgc.ac.cn/VFs) was used.

### Pilus island (PI) and gbs2018 genes

Virulence factors related to PI (PI-1, PI-2a, and PI-2b) and highly virulent gene *gbs2018* (*gbsA*, *gbsB,* and *gbsC*) were identified in the assembled genome in CLC Genomic Workbench 22 by searching the primer binding sites as described earlier [11–13]. The sequences recovered were further confirmed through a nucleotide homology search (http://blast.ncbi.nlm.nih.gov/Blast.cgi). Moreover, the sequence diversity of *gbs2018* genes was determined by alignment and phylogenetic analysis on Mega 11 [14].

### Molecular serotyping

Molecular serotyping was performed based on *cps* gene sequences as described earlier [15]. The nucleotide sequences of CPS*-*encoding clusters were extracted from the *S. agalactiae* sequences and compared to the previously reported nine distinct types of *cps* on Easyfig 2.2 software to determine the sequence heterogeneity.

## Results and Discussion

### Characteristics of the genomes

**Figure 1.**
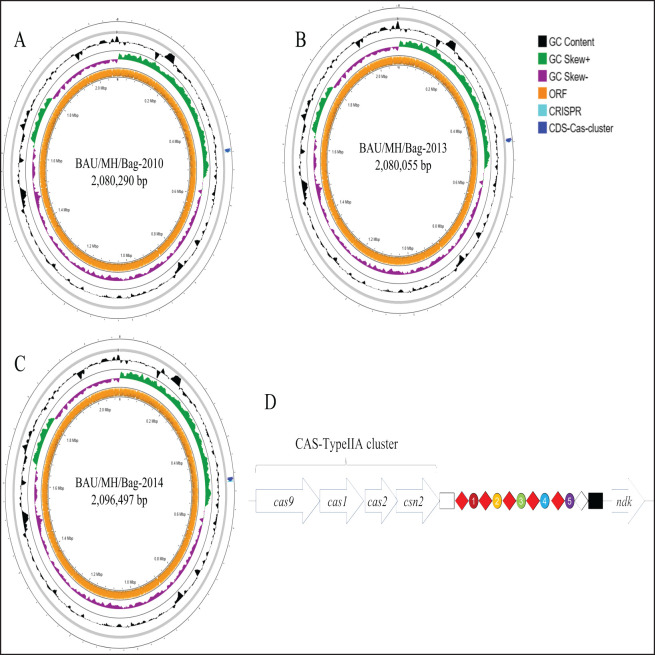
Circular view of the *S. agalactiae* isolated from Bangladesh (BD). This figure shows the distribution of ORFs and CRISPR-Cas systems in the circular view (A–C). The linear view of the Cas-cluster in with the distribution of the *cas* genes, leader sequence (empty rectangle), conserved repeats (red diamond), terminal repeat (white diamond), spacers (different colored ovoids), and the trailer sequence (black rectangle) in *S. agalactiae* genomes 2010, 2013 and 2014 is shown in panel D. Circular view of the genome and other systems were visualized on Proksee tools (https://proksee.ca/).

The *S. agalactiae* isolates 2010, 2013, and 2014 carried genomes of 2,080,290, 2,080,055, and 2096497 bp, respectively, with a 35.2% GC content (Accession no. JAOTOP000000000, JAOTOQ000000000, JAOTOR000000000) (Table 1). No plasmid-like sequences were identified in the genomes, but they contained incomplete and intact phages as well as insertion sequences of different families (Tables 1 and 2). The general genomic features revealed in this study align with those of *S. agalactiae* reported earlier [16]. CRISPR/Cas analysis with CRISPR/Cas Finder (https://proksee.ca/) identified a Type-IIA CRISPR array with an associated CAS cluster containing four gene signatures: cas9-cas1-cas2-csn2). The array consisted of 5 identical repeats typical to that reported earlier (5'-GTT TTA GAG CTG TGC TGT TTC GAA TGG TTC CAA AAC-3') [17] and a non-consensus terminal repeat (Fig. 1D). Spacer sequence analysis revealed typical 30 bp spacers, with two spacers (no. 1 and 2) related to prophages and one (no. 3) with *dnaC*, while the origin of spacers 4 and 5 could not be identified through BLAST search. The Type-IIA CRISPR-cas system is ubiquitous in *S. agalactiae* and is known as a fully functional CRISPR-Cas system. This system is required for the virulence regulation in *S. agalactiae* and provides defense against invading genetic elements like phages, plasmids, and transposons [18,19]. Additionally, this system has regulatory effects on the adherence, invasiveness, and biofilm formation by *S. agalactiae* [20].

### MLST and close neighbors

**Figure 2.**
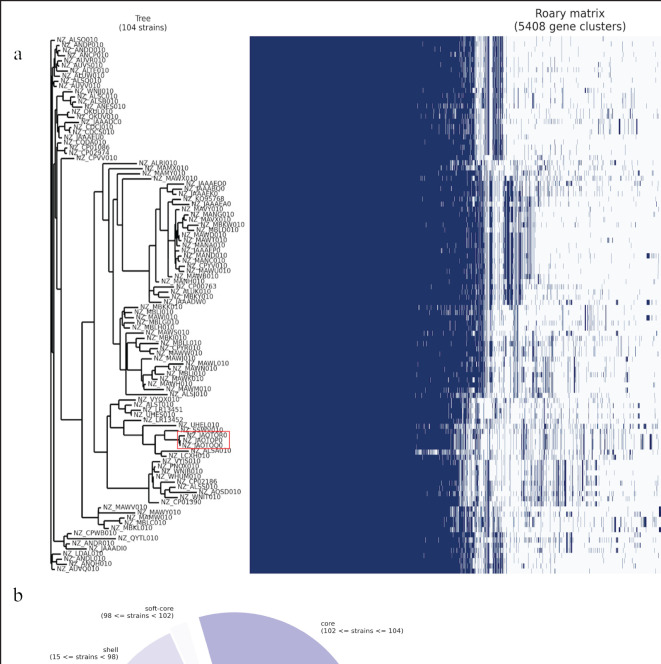
Pangenome analysis of the *S. agalactiae* isolated from Bangladesh (BD). (a) pangenome-based (gene presence and absence) gene clustering matrix of BD (enclosed in red boxes) and isolates from different parts of the world, (b) breakdown of genes in *S. agalactiae* isolates. The figures were prepared with the data obtained from Roary Pangenome analysis using the roary_plots.py script.

**Figure 3.**
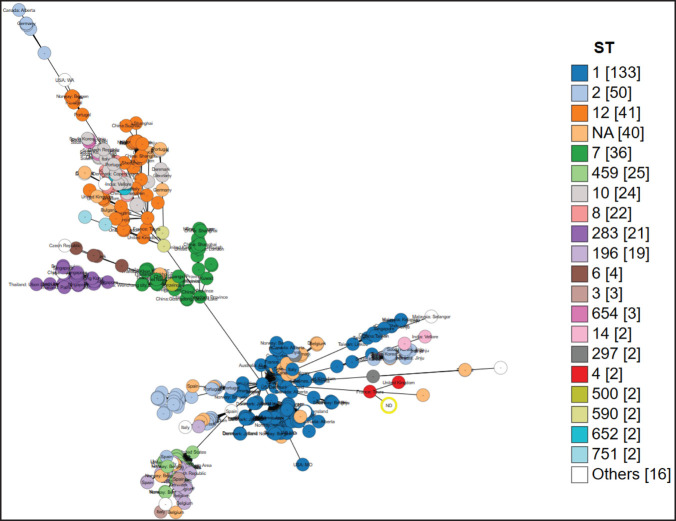
Phylogenetic relationship of *S. agalactiae* isolated in this study and strains reported from different part of the world. The grape tree was prepared on BacWGSTdb website (http://bacdb.cn/BacWGSTdb/) based on core genome multilocus sequence typing (cgMLST) analysis of *S. agalactiae* with SNP and MLST threshold set to 1,000. Different STs are denoted with range of color schemes and the circle highlighted in yellow indicates isolates described in this study.

The *S. agalactiae* isolates belonged to ST4 with unknown clonal complexes through analysis using the PubMLST database. Pan-genome analysis with 104 genomes clustered the isolates 2013, 2014, and 2015 together with minor genetic variations (Fig. 2a), suggesting possible clonality. The occurrence of a clonal strain in the same herd is not impossible. The isolates were closely clustered with human and cow isolates on pan-genome analysis (Fig. 2a), and source tracking through cgMLST analysis revealed close relationships with *S. agalactiae* reported in humans from various parts of the world (Fig. 3). The genomes consisted of 1,592 core genes within a cluster of 5,408 genes identified in the 104 *S. agalactiae* genomes (Fig. 2b). Although the genomes clustered with isolates from humans and animals, the closest isolates were from human vaginal samples reported from France and the United Kingdom, which differ by 84 and 156 alleles, respectively. The closest isolates from cow’s milk differed by 613 alleles, suggesting that the study isolates have zoonotic potential. Previous studies have shown that *S. agalactiae* belongs to different STs with various host specificities [21]. Common strains found in dairy farms around the world are associated with the bovine-adapted ST103, ST568, ST67, ST301, ST313, and ST570 [22], while human isolates belong to ST1, ST7, ST8, ST10, ST12, ST17, ST19, ST23, ST24, ST28, ST110, ST182, ST337, and ST484 [4,23]. The occurrence of ST4 in humans and animals was not readily reported in previous studies and is documented here for the first time in Bangladesh from cases of clinical mastitis in cattle. The close genetic relatedness with both human and animal strains highlights the zoonotic potential of the *S. agalactiae* strains analyzed in this study.

**Table 1.** Characteristics of the *Streptococcus agalactiae* genomes described in this study.

	BAU/MH/Bag-2010	BAU/MH/Bag-2013	BAU/MH/Bag-2014
Bioproject ID	PRJNA879949	PRJNA879959	PRJNA879961
Accession No.	JAOTOP000000000	JAOTOQ000000000	JAOTOR000000000
Genome size (bp)	2080290	2080055	2096497
GC%	35.2	35.2	35.2
No. of contigs	18	20	20
Longest contig (bp)	808064	808066	807742
Mean contig size (bp)	115571.7	104002.8	104824.9
N50	534857	534857	534364
L50	2	2	2
CDS (total)	2077	2074	2097
RNAs	41 [rRNAs: 1, 1, 1 (5S, 16S, 23S); tRNAs: 35; ncRNAs: 3]	41 [rRNAs: 1, 1, 1 (5S, 16S, 23S); tRNAs: 35; ncRNAs: 3]	41 [rRNAs: 1, 1, 1 (5S, 16S, 23S); tRNAs: 35; ncRNAs: 3]
CRISPR arrays	1 (Type IIA) - typical	1 (Type IIA) – typical	1 (Type IIA) – typical
IS elements (no. of sites)	IS3 (4), unknown (5)	IS3 (4), unknown (5)	IS3 (4), unknown (5)
ARGs (CARD analysis)	*mreA*, *mprF*, *vanT*, *vanY, pbp1a, pbp1b, pbp2a, pbp2b, pbp2X, *BAUMH_00180*	*mreA*, *mprF*, *vanT*, *vanY, pbp1a, pbp1b, pbp2a, pbp2b, pbp2X, *BAUMH1_03820*	*mreA*, *mprF*, *vanT*, *vanY, pbp1a, pbp1b, pbp2a, pbp2b, pbp2X, tet(M), *BAUMH2_00180*
Sequence type (ST)	ST4	ST4	ST4
Virulence genes	44	44	44

*CDS encoding for aminoglycoside 6-adenyltransferase conferring resistance to aminoglycosides; ARGs, antimicrobial resistance genes.

**Table 2.** Prophages identified in the *Streptococcus agalactiae* genomes.

Strain	Region	Length	Completeness	CDS	Possible phage	GC%
2010	1	29.7Kb	Incomplete	36	PHAGE_Strept_phiARI0131_2_NC_031941	43.20
	2	24.9Kb	Incomplete	8	PHAGE_Clostr_phiCD27_NC_011398	35.79
	3	50.2Kb	Intact	62	PHAGE_Strept_Str_PAP_1_NC_028666	36.42
2013	1	29.7Kb	Incomplete	36	PHAGE_Strept_phiARI0131_2_NC_031941	43.20
	2	24.9Kb	Incomplete	8	PHAGE_Clostr_phiCD27_NC_011398	35.79
	3	50.2Kb	Intact	62	PHAGE_Strept_315.3_NC_004586	36.42
2014	1	29.7 Kb	Incomplete	36	PHAGE_Strept_phiARI0131_2_NC_031941	43.20
	2	24.9 Kb	Incomplete	8	PHAGE_Clostr_phiCD27_NC_011398	35.79
	3	54.3Kb	Intact	65	PHAGE_Strept_315.3_NC_004586	36.75

**Table 3.** Distribution of pilus island and gbs2018 genes in the *Streptococcus agalactiae* isolates.

Isolate ID	Pilus Island genes			*gbs2018* genes		
	PI-1	PI-2a	PI-2b	A	B	C
BAU/MH/Bag-2010	+	−	+	+	+	−
BAU/MH/Bag-2013	+	−	+	+	+	−
BAU/MH/Bag-2014	+	−	+	+	+	−

+, positive, −, negative.

### AMR and virulence factors

**Figure 4.**
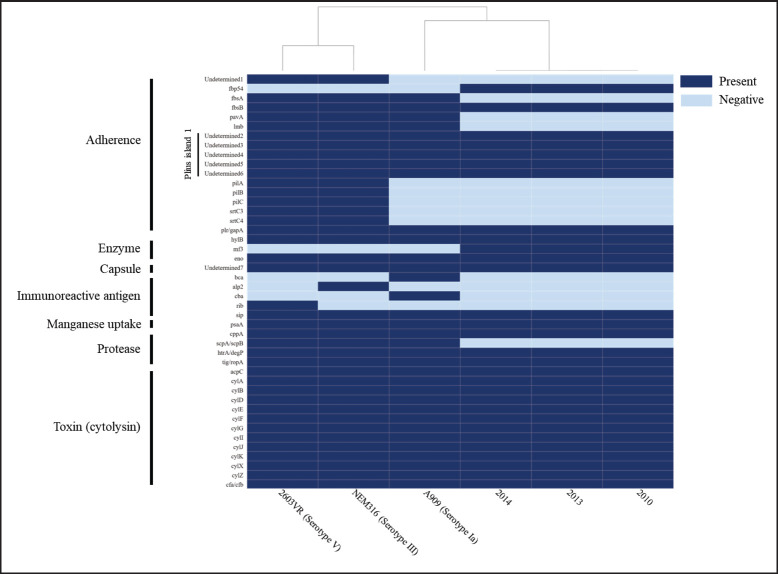
Heat map of the virulence factors present or absent in the *S. agalactiae* genomes from Bangladesh. The virulent genes were identified using VFanalyzer on virulence factor database (VFDB) (www.mgc.ac.cn/VFs). The isolate ID is on the *x*-axis and the virulence genes/ factors are on the y-axis. Isolates from Bangladesh are marked with an underline. The figure was prepared on DISPLAYR (https://southeastasia.displayr.com/) using default parameters and dendrogram appearance.

*Streptococcus agalactiae* harbors various virulence factors such as toxins (*β*-hemolysin/cytolysin and CAMP factor), adhesion and invasion proteins (FbsA, FbsB, *α*C protein, HylB hyaluronidase, and Rib proteins), resistance elements against antibacterial peptides (*β*C protein), and mechanisms to evade the host immune systems (production of C5a peptidase and CspA serine protease) [3]. The *S. agalactiae* genomes analyzed in this study consisted of 44 virulence-related genes, including those associated with adherence (*fbp*, *fbsB*, PI-1), capsule biogenesis, enzymes (*hylB*, *eno*), immunoreactive antigens (*sip*), protease (*htr*A/*deg*P), and toxins (*cylABDEFGIJKXZ*, *cfa*/*cfb*), production, which are considered as the key virulence factors in this bacterium (Fig. 4). These findings indicated that the study strains have the potential to cause mammary gland inflammation and persist in the intra-mammary environment, thereby contributing to mastitis.

In addition to virulence genes, AMR in *S. agalactiae* poses an additional challenge to controlling mastitis as well as a critical concern to public health [1,2,24]. The study strains possess putative genes encoding resistance to aminoglycosides (orf-BAUMH_00180, BAUMH1_03820, and BAUMH2_00180 of strains 2010, 2013, and 2014, respectively), glycopeptides (*vanT, vanY*), macrolides (*mreA*), peptides (*mprF*), *β*-lactams, and penicillins (*pbp*). The genome 2014 also carried the *tet(M)* gene in addition to that mentioned above (Table 1). The genetic profiles correspond to the observed resistance patterns [8]. However, a difference in the phenotypic resistance was evident between the isolates despite carrying a similar set of resistance genes, highlighting the complexity of AMR. The observed difference might be attributed to the gene expression level, additional resistance mechanisms, gene mutations, and environmental factors. Further genomic and transcriptomic analyses would be necessary to elucidate the underlying cause of these differences. This understanding is crucial to developing effective treatment strategies and mitigating the impact of AMR.

### Characteristics of PI and gbs2018 genes

Pilus is an important component of *S. agalactiae* virulence, facilitating adhesion and attachment to host cells and serving as a potential vaccine target. In *S. agalactiae,* three PIs have been described, namely PI-1, PI-2a, and PI-2b. *S. agalactiae* described in this study carried two alleles, PI-1 and PI-2b (Table 3). According to a previous study [13], isolates with PI-1 + PI-2b are frequent in invasive diseases, indicating the study isolates belonged to the invasive subtype. Occurrences of PI-2b have been reported in both human and animal strains with predominance in animal strains [13,23,25,26], and its presence in dairy farms in China and Pakistan suggests its significance in this region [22,27]. Considering the predominance of PI-2b, this study supports the potential of PI-2b protein-based vaccines for *S. agalactiae* mastitis in both humans and animals, particularly bovines.

**Figure 5.**
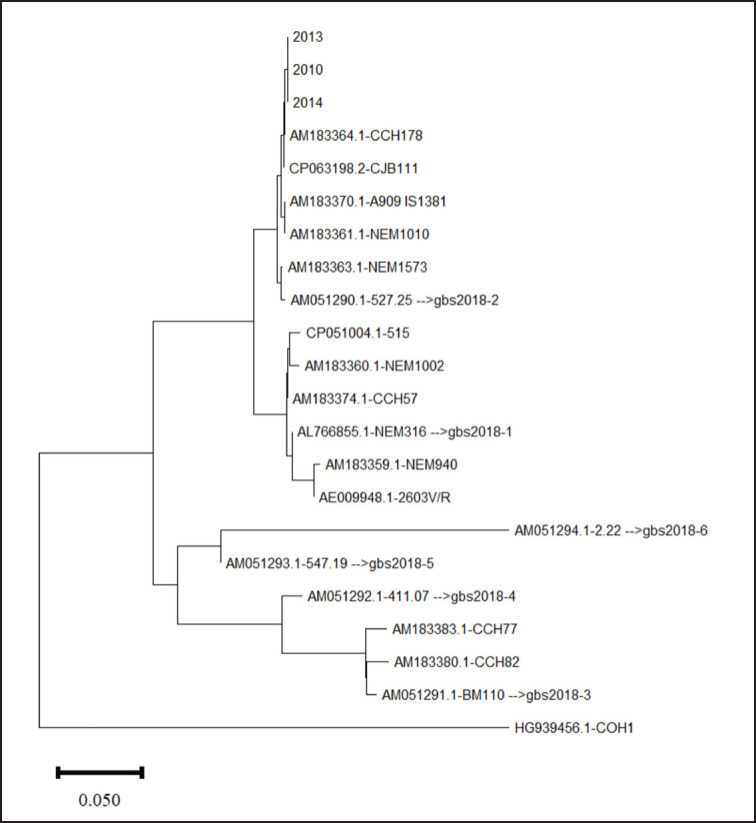
Diversity of *gbs2018* gene alleles in the *S. agalactiae* described in this study. Nucleotide sequences of *gbs2018* gene alleles were downloaded from GenBank and the evolutionary tree was constructed using the Neighbor-Joining method. The evolutionary distances were computed using the *p*-distance method and are in the units of the number of base differences per site. This analysis involved 22 nucleotide sequences. All ambiguous positions were removed for each sequence pair (pairwise deletion option). There were a total of 2836 positions in the final dataset. Evolutionary analyses were conducted in MEGA11 [15].

The *gbs2018* gene encodes a surface adhesin associated with pathogenic GBS strains in humans, animals, and fish. So far, six variants of *gbs2018* have been identified with specific host affinities. Variants *gbs2018-1* to *3* and *5* were reported from humans and animals, while *gbs2018-4* and *gbs2018-6* are found in cattle and fish, respectively [28]. Although comprehensive information on the association of different variants with the virulence of *S. agalactiae* is lacking, variant *gbs2018-3* is associated with hyper-virulent ST17 strains reported from humans [29]. The *gbs2018-3* was crucial to the hyper-virulence of GBS ST17 clones for adherence and translocation through the intestinal and blood-brain barriers [13]. The study isolates carried *gbs2018* A and B genes (Table 3), and the gene sequences were clustered with *gbs2018-2* (Fig. 5), a variant reported from a wide range of hosts including human, bovine, canine, feline, and rodents, indicating the potential of the study isolates to adapt in a wide range of hosts and the high potential of transmission between hosts [28].

### Molecular serotype

**Figure 6.**
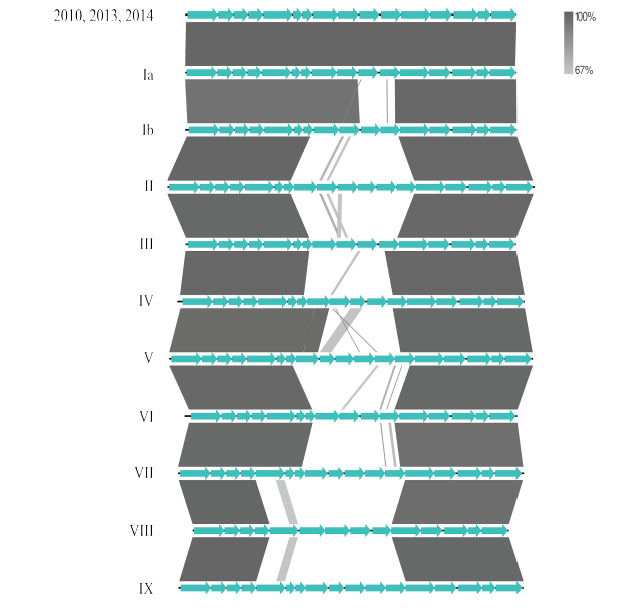
Molecular serotyping of *S. agalactiae* isolated in this study based on capsular polysaccharide (cps) heterogeneity. Nucleotide sequences of *cps* Ia (LT671983), Ib (LT671984), II (LT671985), III (LT671986), IV (LT671987), V (LT671988), VI (LT671989), VII (LT671990), VIII (LT671991) and IX (LT671992) were downloaded from the GenBank and the linear comparison was performed on Easyfig 2.2. This figure shows the identity of nucleotide sequences of genes associated with capsular polysaccharides.

Molecular serotyping is important for the epidemiological discrimination of GBS, which involves the analysis of *cps* gene sequences. Previous studies indicated that capsular serotypes may vary among different populations and geographical locations. A total of ten capsular serotypes have been reported in *S. agalactiae*, with serotypes Ia, Ib, III, and V being more prevalent in the USA and Europe, and serotypes Ia, Ib, II, III, IV, and V are commonly encountered in the South and Southeast regions of Brazil [30]. On the other hand, serotypes VI to IX are sparsely described [31,32]. Thus, determining the capsular serotype is important from an epidemiological point of view as well as for developing a vaccine targeting this potential virulence determinant. Linear comparison on EasyFig revealed that the *cps* gene clusters in the study isolates were identical to *cps*-Ia (Fig. 6), aligning with the lineage described in different countries and associated with invasive diseases [33].

### Limitations

The study was limited to three isolates from a single dairy farm in Bangladesh, restricting its ability to provide a comprehensive view of the molecular characteristics of *S. agalactiae* circulating in the region. Additionally, the genetic characteristics were not validated with phenotypic experiments, limiting the interpretation of genotype-phenotype associations. Thus, further studies with more isolates from different areas of Bangladesh and phenotypic validations are necessary to ascertain the virulence potential and develop effective strategies to control *S. agalactiae*-associated mastitis and its dissemination to other environmental components, including humans.

## Conclusion

Despite these limitations, this study explored crucial genetic information in *S. agalactiae* isolated from bovine clinical mastitis in Bangladesh. The isolates belonged to ST4 with a novel clonal complex, carrying virulence genes essential for persistence and pathogenesis in the intra-mammary environment. The isolates belonged to serotype Ia and possess PI-1 and PI-2b, and the *gbs2018-2* gene variant, suggesting they are invasive subtypes capable of adapting to diverse hosts, including humans, animals, and rodents. The isolates also harbored multiple AMR genes, highlighting their pathogenic potential and the importance of selecting appropriate antimicrobials for treatment. This study provides a snapshot of *S. agalactiae* genotypes present in the dairy population of Bangladesh, which is crucial for further studies and suggests vaccine development or importing protein-based vaccines developed against specific virulence determinants such as PI-2b to control *S. agalactiae* mastitis in cattle in Bangladesh.

## Data Availability

The supplementary data can be accessed on request to the author.

## List of abbreviations

AMR, antimicrobial resistance; ARGs, antimicrobial resistance genes; CARD, comprehensive antibiotic resistance database; CCs, clonal complexes; cgMLST, core genome multilocus sequence typing; GBS, Group B Streptococcus; GC, guanine-cytosine; PI, pilus island; WGS, whole genome sequencing; VFDB, virulence factor database; VFs, virulence factors.

## References

[1] Hernandez L, Bottini E, Cadona J, Cacciato C, Monteavaro C, Bustamante A (2021). Multidrug resistance and molecular characterization of *Streptococcus agalactiae* isolates from dairy cattle with mastitis. Front Cell Infect Microbiol.

[2] Han G, Zhang B, Luo Z, Lu B, Luo Z, Zhang J (2022). Molecular typing and prevalence of antibiotic resistance and virulence genes in *Streptococcus agalactiae* isolated from Chinese dairy cows with clinical mastitis. PLoS One.

[3] Wataradee S, Boonserm T, Samngamnim S, Ajariyakhajorn K (2024). Characterization of virulence factors and antimicrobial susceptibility of *Streptococcus agalactiae* associated with bovine mastitis cases in Thailand. Animals.

[4] Furfaro LL, Chang BJ, Payne MS (2018). Perinatal *Streptococcus agalactiae* epidemiology and surveillance targets. Clin Microbiol Rev.

[5] Pitts SI, Maruthur NM, Langley GE, Pondo T, Shutt KA, Hollick R (2018). Obesity, diabetes, and the risk of invasive group B streptococcal disease in nonpregnant adults in the United States. Open Forum Infect Dis.

[6] Chaguza C, Jamrozy D, Bijlsma MW, Kuijpers TW, van de Beek D, van der Ende A (2022). Population genomics of Group B *Streptococcus* reveals the genetics of neonatal disease onset and meningeal invasion. Nat Commun.

[7] Boonyayatra S, Wongsathein D, Tharavichitkul P (2020). Genetic relatedness among *Streptococcus agalactiae* isolated from cattle, fish, and humans. Foodborne Pathog Dis.

[8] Hassan J, Bag MAS, Ali MW, Kabir A, Hoque MN, Hossain MM (2023). Diversity of *Streptococcus* spp. and genomic characteristics of *Streptococcus uberis* isolated from clinical mastitis of cattle in Bangladesh. Front Vet Sci.

[9] The Galaxy Community (2022). The Galaxy platform for accessible, reproducible and collaborative biomedical analyses: 2022 update. Nucleic Acids Res.

[10] Page AJ, Cummins CA, Hunt M, Wong VK, Reuter S, Holden MTG (2015). Roary: rapid large-scale prokaryote pan genome analysis. Bioinformatics.

[11] Lamy MC, Dramsi S, Billoët A, Réglier-Poupet H, Tazi A, Raymond J (2006). Rapid detection of the ‘highly virulent’ group B *streptococcus* ST-17 clone. Microb Infect.

[12] Tazi A, Disson O, Bellais S, Bouaboud A, Dmytruk N, Dramsi S (2010). The surface protein HvgA mediates group B *streptococcus* hypervirulence and meningeal tropism in neonates. J Exp Med.

[13] Madzivhandila M, Adrian PV, Cutland CL, Kuwanda L, Madhi SA, The PoPS Trial Team (2013). Distribution of pilus islands of group B *streptococcus* associated with maternal colonization and invasive disease in South Africa. J Med Microbiol.

[14] Tamura K, Stecher G, Kumar S (2021). MEGA 11: molecular evolutionary genetics analysis version 11. Mol Biol Evol.

[15] Poyart C, Tazi A, Réglier-Poupet H, Billoët A, Tavares N, Raymond J (2007). Multiplex PCR assay for rapid and accurate capsular typing of group B streptococci. J Clin Microbiol.

[16] de Aguiar EL, Mariano DC, Viana MV, Benevides LJ, de Souza Rocha F, de Castro Oliveira L (2016). Complete genome sequence of *Streptococcus agalactiae* strain GBS85147 serotype of type Ia isolated from human oropharynx. Stand Gen Sci.

[17] Lopez-Sanchez MJ, Sauvage E, Da Cunha V, Clermont D, Ratsima Hariniaina E, Gonzalez-Zorn B (2012). The highly dynamic CRISPR1 system of *Streptococcus agalactiae* controls the diversity of its mobilome. Mol Microbiol.

[18] Schelling MA, Sashital DG (2020). Bacterial Immunity: an adaptable defense. eLife.

[19] Dong Y, Ma K, Cao Q, Huang H, Nie M, Liu G (2021). CRISPR-dependent endogenous gene regulation is required for virulence in piscine *Streptococcus agalactiae*. Emerg Microb Infect.

[20] Nie M, Dong Y, Cao Q, Zhao D, Ji S, Huang H (2022). CRISPR contributes to adhesion, invasion, and biofilm formation in *Streptococcus agalactiae* by repressing capsular polysaccharide production. Microbiol Spectr.

[21] Sirimanapong W, Phuoc NN, Crestani C, Chen S, Zadoks RN (2023). Geographical, temporal and host-species distribution of potentially human-pathogenic group B *Streptococcus* in aquaculture species in Southeast Asia. Pathogens.

[22] Yang Y, Liu Y, Ding Y, Yi L, Ma Z, Fan H (2013). Molecular characterization of *Streptococcus agalactiae* isolates from bovine mastitis and human infections. BMC Microbiol.

[23] Pang M, Sun L, He T, Bao H, Zhang L, Zhou Y (2017). Molecular and virulence characterization of highly prevalent *Streptococcus agalactiae* circulated in bovine dairy herds. Vet Res.

[24] Zhuk Y, Zaritskyi R, Dreval D, Derkach S, Kovpak V, Masalovych Y (2022). Antimicrobial susceptibility of mastitis pathogens of dairy cows in Ukraine. Potrav Slovak J Food Sci.

[25] Margarit I, Rinaudo CD, Galeotti CL, Maione D, Ghezzo C, Buttazzoni E (2009). Preventing bacterial infections with pilus-based vaccines: the group B *Streptococcus* paradigm. J Infect Dis.

[26] Nabavinia M, Khalili MB, Sadeh M, Eslami G, Vakili M, Azartoos N (2020). Distribution of Pilus island and antibiotic resistance genes in *Streptococcus agalactiae* obtained from vagina of pregnant women in Yazd, Iran. Iran J Microbiol.

[27] Leghari A, Lakho SA, Khand FM, Bhutto Kur R, Lone SQ, Aleem MT (2023). Molecular epidemiology, characterization of virulence factors and antibiotic resistance profile of *Streptococcus agalactiae* isolated from dairy farms in China and Pakistan. J Integr Agric.

[28] Delannoy CMJ (2013). Host adaptation of aquatic *Streptococcus agalactiae*. https://core.ac.uk/download/pdf/18538301.pdf.

[29] Santi I, Scarselli M, Mariani M, Pezzicoli A, Masignani V, Taddei A (2007). BibA: a novel immunogenic bacterial adhesin contributing to group B *Streptococcus* survival in human blood. Mol Microbiol.

[30] Dutra VG, Alves VM, Olendzki AN, Dias CA, de Bastos AF, Santos GO (2014). *Streptococcus agalactiae* in Brazil: serotype distribution, virulence determinants and antimicrobial susceptibility. BMC Infect Dis.

[31] Williams AN, Croxen MA, Demczuk WHB, Martin I, Tyrrell GJ (2023). Genomic characterization of emerging invasive *Streptococcus agalactiae* serotype VIII in Alberta, Canada. Eur J Clin Microbiol Infect Dis.

[32] Slotved HC, Møller JK, Khalil MR, Nielsen SY (2021). The serotype distribution of *Streptococcus agalactiae* (GBS) carriage isolates among pregnant women having risk factors for early-onset GBS disease: a comparative study with GBS causing invasive infections during the same period in Denmark. BMC Infect Dis.

[33] Martins ER, Melo-Cristino J, Ramirez M, Portuguese group for the study of streptococcal infections (2012). Dominance of serotype Ia among group B Streptococci causing invasive infections in nonpregnant adults in Portugal. J Clin Microbiol.

